# Zebrafish Avatars of rectal cancer patients validate the radiosensitive effect of metformin

**DOI:** 10.3389/fonc.2022.862889

**Published:** 2022-09-28

**Authors:** Bruna Costa, Laura M. Fernandez, Oriol Parés, Ricardo Rio-Tinto, Inês Santiago, Mireia Castillo-Martin, Amjad Parvaiz, Rita Fior

**Affiliations:** ^1^ Champalimaud Research, Champalimaud Foundation, Lisbon, Portugal; ^2^ Colorectal Surgery Department, Champalimaud Clinical Centre, Champalimaud Foundation, Lisbon, Portugal; ^3^ Radiation Oncology Department, Champalimaud Clinical Centre, Champalimaud Foundation, Lisbon, Portugal; ^4^ Gastroenterology Department, Champalimaud Clinical Centre, Champalimaud Foundation, Lisbon, Portugal; ^5^ Radiology Department, Champalimaud Clinical Centre, Champalimaud Foundation, Lisbon, Portugal; ^6^ Pathology Service, Champalimaud Clinical Centre, Champalimaud Foundation, Lisbon, Portugal

**Keywords:** rectal cancer, metformin, radiotherapy, neoadjuvant chemoradiation, zebrafish Avatars

## Abstract

Neoadjuvant chemoradiation (nCRT) followed by surgery represents the standard of care in patients with locally advanced rectal cancer. Increasing radiotherapy (RT) doses and chemotherapy cycles with 5FU have been associated with increased rates of complete response, however these strategies imply significant toxicity. In the last years, epidemiologic findings have demonstrated that metformin is associated with significantly higher rates of pathological complete response to nCRT. Also, pre-clinical studies using cell lines provide evidence for the radiosensitive effect of metformin. However, no studies have been performed using rectal cancer patient samples to test this radiosensitive effect of metformin and compared it to the standard 5FU. Here, we designed an experimental study to compare both radiosensitizers in the zebrafish xenograft model (zAvatar), using rectal cancer surgical specimens and diagnostic biopsies. Patient zAvatars confirmed that metformin has indeed a powerful *in vivo* radiosensitizer effect, similar to 5FU. Our work confirms that metformin constitutes a promising less toxic alternative to the standard 5FU, which could be game changing in elderly/frail patients to optimize tumor regression.

## Introduction

ESMO guidelines for neoadjuvant therapy in rectal cancer relies on two approaches: long course neoadjuvant chemoradiotherapy (LC-CRT), consisting of 25–28 fractions of 1.8–2Gy with concomitant administration of 5FU; and short-course radiotherapy (scRT), consisting of 5Gy over five consecutive days (5×5Gy), usually followed by 5FU infusion (1).

The benefit of irradiating the rectum before surgery has been associated with high rates of tumor shrinkage, resulting in better local control of the disease (2). In some cases, tumor response to radiotherapy (RT) leads to a complete disappearance of the tumor with excellent survival rates (3, 4). In addition, patients with endoscopic and radiological evidence of complete tumor response after nCRT may follow a non-operative management approach (Watch & Wait) avoiding all major consequences of surgery with similar oncological outcomes (5–7). In this scenario, two issues become most relevant: i) increase tumor response rates to radiotherapy to avoid surgery-related morbidity; ii) identify patients who will not respond to nCRT to avoid radiation-related morbidity and proceed immediately to surgery. Escalating radiation therapy doses and additional chemotherapy cycles have been associated with increased rates of complete response to nCRT (8–10). However, these strategies may increase toxicity and morbidity rates (11, 12).

Interestingly, some clinical studies associated the use of metformin (MET), a drug used for diabetes treatment, with higher rates of complete tumor response to nCRT in patients with rectal cancer (13, 14). In addition, experimental studies *in vitro* and *in vivo* using colorectal cancer (CRC) cell lines and mouse models, have demonstrated that association of MET with RT was as efficient as the classical association of RT with 5FU in impairing tumor growth, suggesting its use as an alternative radiosensitizing agent (15). However, to our knowledge no studies have yet been performed using rectal cancer patient samples *in vivo*.

In the last years a novel *in vivo* model has been developed, the zebrafish Patient Derived Xenograft -”zAvatar” model for personalized medicine (16–18). This assay relies on the injection of fluorescently labelled tumor cells into 2 days post fertilization (dpf) zebrafish embryos and accessing tumor behavior and response to anti-cancer therapy after 3-4 days. zAvatars offer speed, single-cell resolution, large numbers of transplants and evaluation of crucial cancer hallmarks, only possible due to the high genetic conservation between the human and the zebrafish genome. Recently, our group developed a protocol to assess *in vivo* radiotherapy response. We showed that is possible to distinguish radiosensitive from radioresistant tumors in zebrafish xenografts, even in polyclonal tumors, in just 4 days (19).

Here, we present a short report where we evaluated the radiosensitizing effect of metformin *in vivo* using zAvatars. We used not only CRC cell lines but also patient samples without *in vitro* expansion: rectal cancer surgical specimens and, importantly, rectal tumor diagnostic biopsies, which is technically challenging due to the small amount obtained by endoscopy. Our results provide further evidence of the radiosensitive effect of metformin in rectal cancer. Patient zAvatars tumors showed a diversity of responses but in general MET was beneficial in sensitizing the tumors to radiotherapy. This diversity of responses underlines the necessity for a personalized test prior to treatment, a clear unmet need in the oncology field.

## Material and methods

### Animal care and handling

*In vivo* experiments were performed using zebrafish (Danio rerio), which were maintained and handled in accordance with European Animal Welfare Legislation, Directive (2010/63/EU), and Champalimaud Fish Platform. The study and procedures were approved by the Ethical Committee and Portuguese institutional organizations: ORBEA (Animal Welfare and Ethics Body) and DGAV (Directorate General for Food and Veterinary).

### Zebrafish lines

Experiments were performed using transparent Nacre, which has complete lack of melanocytes due to a mutation in the gene encoding the *mitfa* gene (20), and *Tg(Fli1:eGFP)*, which allows the visualization of blood and lymphatic vessels, through the expression of eGFP linked to fli1 (endothelial marker) promoter (21).

### Patient samples

The study was approved by the Ethics Committee of the Champalimaud Foundation. Rectal cancer patient samples were provided by Champalimaud Clinical Center’s (CCC) Digestive Unit, after signed informed consent. Patients’ inclusion criteria were: pathology confirmed adenocarcinoma of the rectum (tumor below 15 cm from the anal verge). Exclusion criteria: previous diagnosis of diabetes, under metformin treatment. Tissue from surgically resected rectal cancer samples and biopsies were collected in culture media containing a mixture of antibiotics and antifungals (**Supplementary Table S1**) and cryopreserved until injection. For microinjection, samples were thawed and minced in Mix1 (**Supplementary Table S1**) with subsequent mechanical tissue fragmentation and centrifugation (250xg, 4 min). The remaining tissue fragments were enzymatically digested in Mix2 (**Supplementary Table S1**), passed through a 70-um cell strainer and labeled at 37°C. Tumor cells were resuspended in Mix1 and checked for viability with Trypan Blue dye exclusion.

### Human colorectal cancer cell lines

HCT116 (KRAS^G13D^) and Hke3 (KRAS^WT^) were kindly provided by Angela Relógio (Institute for Theoretical Biology, Berlin). Cell lines were tested for mycoplasma and authenticated through Short Tandem Repeat (STR) profiling.

### Cell culture

Cell lines were expanded and maintained in Dulbecco’s Modified Eagle Medium (DMEM) High Glucose (Biowest) supplemented with 10% (v/v) Fetal Bovine Serum (FBS) (Sigma-Aldrich) and 1% (v/v) Penicillin-Streptomycin 10,000 U/mL (Hyclone). Cells were maintained with a humidified atmosphere at 5% CO2 and 37°C.

### Cell staining

Cells were labeled with Vybrant CM-DiI (Thermo Fisher Scientific) at a concentration of 4μL/mL or with Deep Red (CellTracker, Thermo Fisher Scientific) at a concentration of 1μL/mL. Staining was performed according to manufacturer’s instructions. Cells were resuspended to a final concentration of 0,25 × 10^6^ cells/μL.

### Zebrafish xenograft microinjection

Labelled cells were microinjected using borosilicate glass capillaries under a fluorescence scope (Zeiss Axio Zoom.V16) with a mechanical pneumatic injector attached (Pneumatic Pico pump PV820, World Precision Instruments). Cells were injected into the perivitelline space (PVS) of anesthetized 2dpf zebrafish embryos. After injection, embryos were maintained at 34°C until the end of the experiments in E3 medium. At 1 day post-injection (dpi), zebrafish xenografts were screened regarding the presence or absence of a tumoral mass. Xenografts with severe edema, cells in the yolk sac, cell debris or non-injected zebrafish embryos were discarded (22). At 3 or 4dpi, xenografts were sacrificed, fixed with 4% formaldehyde (Thermo Scientific) at 4°C overnight and preserved at -20°C in 100% methanol (VWR Chemicals). Percentage of tumor implantation was calculated as follows:


$$\text{\% implantation}=\;\frac{\text{n°xenografts at }3\text{ or }4\text{dpi with a tumor mass}}{\text{n°xenografts at }4\text{dpi}}\;x\;100$$

### Xenografts irradiation and drug administration

At 1dpi zebrafish xenografts were randomly distributed into different experimental conditions: control (non-treated and non-irradiated), radiation alone (25Gy), 5FU alone (5FU), metformin alone (MET), radiation+5FU (25Gy+5FU) or radiation+metformin (25Gy+MET). Radiation consisted of a single high dose of 25Gy at 1dpi, as previously described (19). Irradiation procedures and regimens were adapted for zebrafish xenografts by the Champalimaud Foundation Radiation Oncology Department. The 6MV X-rays beams with the corresponding prescription dose (25Gy) were calculated with the same algorithm used in clinical practice (ECLIPSE, Varian Medical System, CA) and was delivered *via* a linear accelerator (Truebeam, Varian Medical Systems, CA). Irradiation was targeted to the center of a defined area of 30x30cm where the 6-well plates with the anesthetized zebrafish were placed. The well plates were positioned with a source-to-surface distance of 100cm. No build up material was needed. Also, at 1dpi, 5FU (4.2 mM) or metformin (5 mM) were administered in E3 during two or three successive days, depending on the experiment. Both solutions were freshly prepared daily. Metformin concentration was calculated after performing a maximum tolerated concentration (MTC) experiment using 0.05mM, 0.5mM, and 5mM of metformin in non-injected zebrafish embryos (data not shown). We observed a phenotype with 0.05mM in Hke3 cells (induction of cell death and decrease in tumor size). However, since no toxic effects were observed in all concentrations, we decided to use the highest to be sure that a lack of response (resistance) is not due to a problem of drug availability.

### Xenografts whole-mount immunofluorescence

Primary antibodies: anti-activated caspase3 (rabbit, Cell Signaling, 1:100, code#9661), anti-human mitochondria (mouse, Merck Millipore, 1:100, cat#MAB1273). Secondary antibodies: Alexa goat anti-rabbit 488 (Molecular Probes, 1:400), anti-mouse 488 (Molecular Probes, 1:400), and anti-mouse 647 (Molecular Probes 1:400) were applied simultaneously with DAPI. Xenografts were mounted with Mowiol.

### Xenografts imaging and quantification

Xenografts were acquired using a Zeiss LSM 710 fluorescence confocal microscope, with a 5μM interval z-stack. Images were analyzed using ImageJ software, using the Cell Counter plugin (23). To assess tumor size, three representative slices of each tumor, from the top (Zfirst), middle (Zmiddle), and bottom (Zlast) were analyzed and a proxy of total cell number of the entire tumor (DAPI nuclei) was estimated as follows:


$$\text{tumor size}=\;\frac{\text{AVG(n°of DAPI cells Zfirst+n°of DAPI cells Zmiddle+n°of DAPI cells Zlast) }x\;total\;number\;of\;slices}{1.5}$$

The number of mitotic figures and activated caspase3 were quantified manually, counting all cells in every slice (from Zfirst to Zlast) and the respective percentages were generated by dividing the values by the tumor size (n° of tumor cells).

### Immunohistochemistry

Formalin-fixed paraffin embedded (FFPE) tissue sections from each patient were used to evaluate the P53 status by immunohistochemistry with the anti-P53 monoclonal antibody (P53-DO7-L-CE, Leica, cat#PA0057), using the Leica Bond Max automated system (Leica Biosystems). A mutant phenotype was considered when a diffuse and intense nuclear staining was observed in the tumor cells (overexpression), whereas a wild-type phenotype consisted in spare and mild nuclear expression.

### KRAS analysis

Mutations in KRAS (NM_004985.4) were determined with the IdyllaTM real-time PCR automatized system (Biocartis).

### Statistical analysis

Statistical analysis was performed using GraphPad Prism 8.0 software. All datasets were challenged by normality tests (D’Agostino and Pearson and the Shapiro–Wilk). A Gaussian distribution was only assumed for datasets that pass both normality tests and were analyzed by an unpaired t-test with Welch’s correction. Datasets without Gaussian distribution were analyzed by unpaired and nonparametric Man-Whitney test. For all the statistical analysis, p value is from a two-tailed test with a confidence interval of 95%. Statistical differences were considered significant whenever p< 0.05 and statistical output was represented by stars as follows: non-significant (ns) > 0.05, *p ≤ 0.05, **p ≤ 0.01, ***p ≤ 0.001 and ****p ≤ 0.0001. All graphs presented the results as average (AVG) ± standard error of the mean (SEM).

## Results

### Metformin has a similar radiosensitive effect to 5FU

In our previous work we developed a single dose radiotherapy protocol of 25Gy to assess radiosensitivity *in vivo* in just 4 days (19). Here, in order to evaluate whether the zebrafish xenograft model is able to reveal the radiosensitizing effect of metformin (MET), we chose two isogenic CRC that we previously characterized: radiosensitive HCT116 and radioresistant Hke3 cells (19). These two cells lines differ in their KRAS status: HCT116 harbor a KRAS^G13D^ mutation whereas the isogenic Hke3 cells are KRAS^WT^ (24).

**Figure 1:**
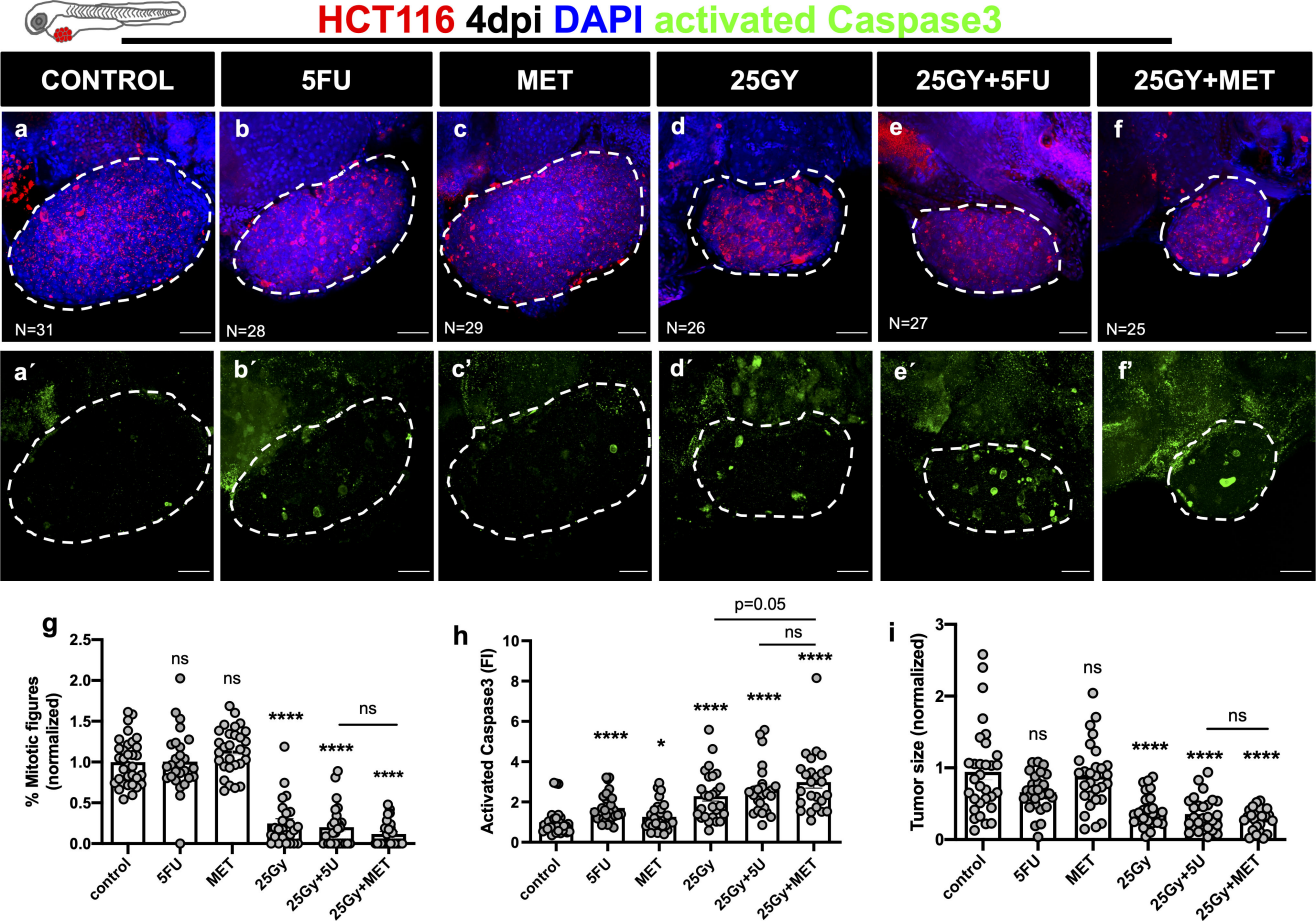
Human CRC cells HCT116 were injected into the PVS of 2dpf zebrafish embryos. At 1dpi xenografts were submitted to 5FU chemotherapy **(B)**, metformin **(C)**, single radiation dose of 25Gy **(D)** or combinations of 25Gy+5FU **(E)** or 25Gy+MET **(F)**. HCT116 xenografts were treated for 3 consecutive days and compared to non-irradiated and non-treated controls **(A)**. Maximum Z projections of activated Caspase3 are shown in green **(A’–F’)**. At 4dpi, cell proliferation (% mitotic figures), apoptosis (% activated Caspase3, green), and tumor size (number of tumor cells, DAPI, blue) were analyzed and quantified (**G–I** respectively). Results are the average of 2 independent experiments and are expressed as mean ± SEM. Each dot represents a xenograft and the total number of xenografts analyzed is indicated in the images **(A–F)**. Dashed white line is delimitating the tumor of each xenograft. Scale bars represent 50µm. Statistical results: (ns) > 0.05, *P≦0.05, ****P≦0.0001. Results were compared with control with exception of those that have a bar to indicate the compared groups.

CRC tumor cells were fluorescently labeled and injected into the periviteline space (PVS) of 2 days post fertilization (dpf) zebrafish embryos. To study *in vivo* the outcomes of radiation combined with metformin and its comparison to 5FU, 6 conditions were tested: control, 5FU, MET, 25Gy, 25Gy+5FU and 25Gy+MET (**Figure 1**). At 1 day post-injection (1dpi) injected embryos were submitted to 25Gy in a single radiation session. According to the condition, this procedure was immediately followed by the addition of 5FU or metformin in E3 medium for three consecutive days, replaced daily. Single 5FU and MET treatments were also delivered for three consecutive days starting at 1dpi. Control refers to non-irradiated and non-treated xenografts. At 4dpi xenografts were processed for immunofluorescence and confocal imaging. The impact of treatment was analyzed by quantifying proliferation (mitotic figures), induction of apoptosis (activated caspase3) and tumor size (number of tumor cells) (**Figure 1**).

In HCT116 xenografts we could not detect any significant difference in proliferation with 5FU treatment (p=0.9412), or MET alone (1.14 fold change, p=0.066) (**Figure 1G**). In contrast, RT alone or in combination with either 5FU or MET strongly impaired proliferation (75%, 80% and 88% reduction respectively, p<0.0001). However, no difference was observed between 25Gy+5FU vs 25Gy+MET (p=0.2295) (**Figure 1G**). In terms of cell death by apoptosis, we observed a clear effect of 25Gy+MET, with a ~3 fold increase in activated caspase3 (p<0.0001), similar to the effect of 5FU (fold increase ~2.60, p<0.0001). Again, we could not detect significant differences between 25Gy+5FU and 25Gy+MET (p=0.3255) conditions (**Figure 1H**).

Regarding tumor size, 5FU or MET single treatments did not led to a significant tumor shrinkage (fold decrease 0.66, p=0.1357 and 0.88, p=0.9298, respectively) (**Figures 1B, C, I**). In contrast, RT alone or in combination with 5FU or MET led to a strong and similar reduction in tumor size (59%, 64% and 72% shrinkage, respectively p<0.0001 for all) (**Figures 1E, F, I**). The difference between 25Gy+5FU and 25Gy+MET was not significant (p=0.4027) (**Figure 1I**).

In summary, our results show that MET has a similar radiosensitization effect as 5FU in HCT116 zebrafish xenografts.

### Metformin is able to sensitize Hke3 xenografts to radiotherapy

**Figure 2:**
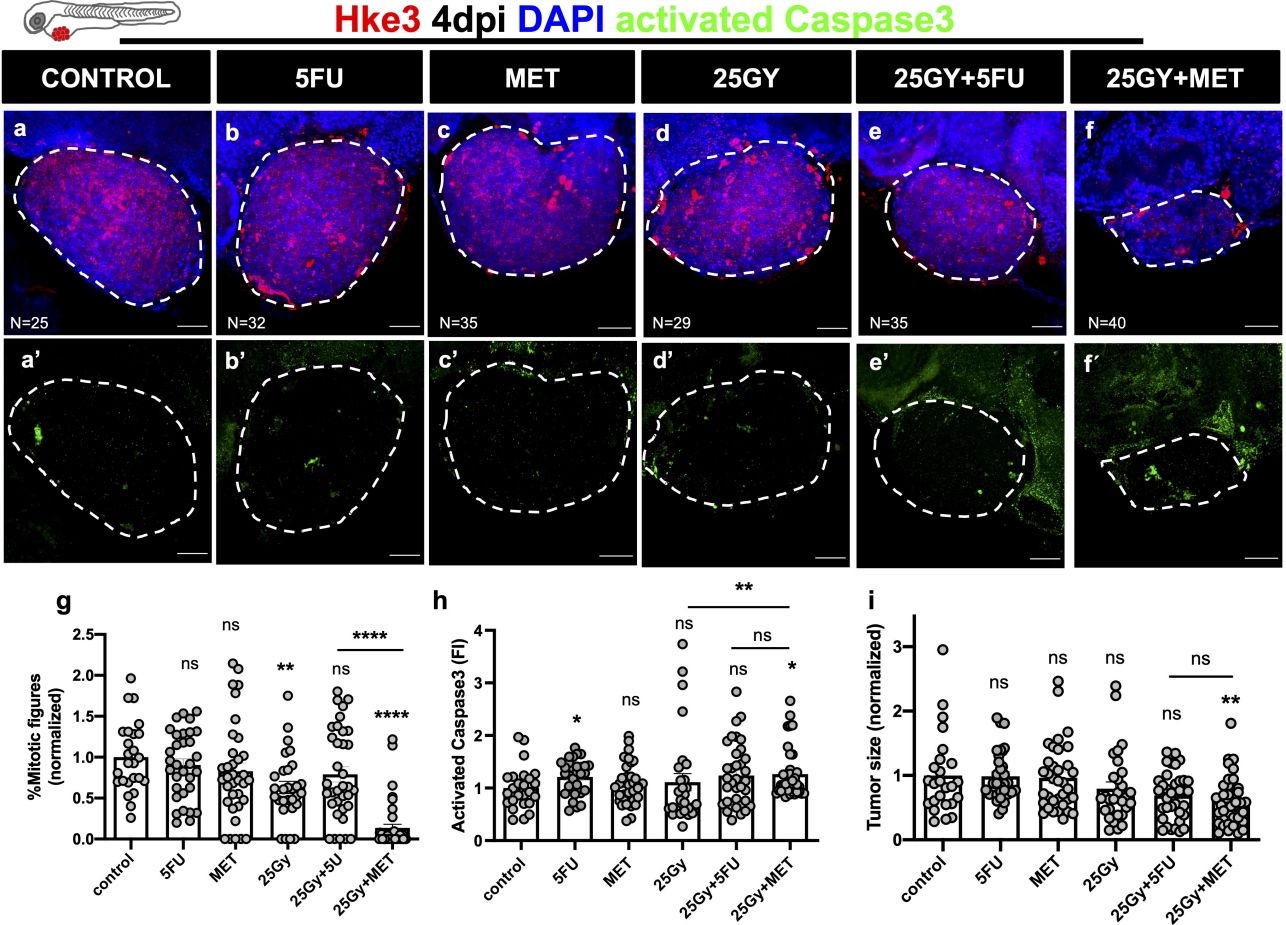
Human CRC cells Hke3 were injected into the PVS of 2dpf zebrafish embryos. At 1dpi xenografts were submitted to 5FU chemotherapy **(B)**, metformin **(C)**, single radiation dose of 25Gy **(D)** or combinations of 25Gy+5FU **(E)** or 25Gy+MET **(F)**. HKe3 xenografts were treated for 3 consecutive days and compared to non-irradiated and non-treated controls **(A)**. Maximum Z projections of activated Caspase3 are shown in green **(A’–F’)**. At 4dpi, cell proliferation (% mitotic figures), apoptosis (% activated Caspase3, green), and tumor size (number of tumor cells, DAPI, blue) were analyzed and quantified (**G–I** respectively). Results are the average of 2 independent experiments and are expressed as mean ± SEM. Each dot represents a xenograft and the total number of xenografts analyzed is indicated in the images **(A–F)**. Dashed white line is delimitating the tumor of each xenograft. Scale bars represent 50µm. Statistical results: (ns) > 0.05, *P≦0.05, ** P≦0.01, ****P≦0.0001. Results were compared with control with exception of those that have a bar to indicate the compared groups.

Next, we generated radioresistant Hke3 xenografts to test whether MET could radiosensitize these refractory cells (25). Six conditions were tested as before: control, 5FU, MET, 25Gy, 25Gy+5FU and 25Gy+MET (**Figures 2A-F**). As expected, RT or its combination with 5FU had no significant effect in inducing apoptosis or tumor shrinkage (**Figures 2D, E, G–I**). Strikingly, combination of 25Gy+MET induced a significant induction of apoptosis (1.27 fold induction, p=0.0139) and reduction of tumor size (40% reduction of tumor size, p=0.030). Importantly, the difference between 25Gy and 25Gy+MET was significant, demonstrating its sensitizer effect over radiation (p=0.031) (**Figure 2H**). Regarding proliferation, combination of RT with MET had a clear synergistic effect and almost completely abolished the proliferation capacity of these cells (86% reduction, p<0.0001) (**Figure 2G** and **Supplementary Figure S1**).

Although we observed a significant reduction in tumor size and proliferation with 25Gy+MET treatment, the impact on apoptosis induction was quite mild. This was puzzling since apoptosis represents one of the major types of cell death induced by ionizing radiation (26) and, therefore, is a very good surrogate for radiosensitivity. Also, in our previous work we showed that apoptosis is our main readout to define sensitivity/resistance to chemo and radiotherapy (18, 19). Therefore, we wondered whether the peak of apoptosis had occurred before. To investigate this, we repeated the same experiment but analyzed the xenografts at 2 timepoints: 3dpi (2 days of treatment) and 4dpi (3 days of treatment). Indeed, we were able to observe an earlier peak of apoptosis at 3dpi (from an AVG of 2.2% in controls to 4.7% in 25Gy+MET, corresponding to 2.13 fold induction of apoptosis, p<0.0001) (**Supplementary Figure S2B**). At this timepoint there is a remarkable sensitizer effect of 25Gy+MET over radiation alone in terms of cell death (p<0.0001) and a strong reduction of tumor size (p=0.016), when comparing these two conditions (**Supplementary Figures S2B, C**). Our results with Hke3 show that, indeed, MET can sensitize even radioresistant tumor cells, and in this case more efficiently than 5FU.

### zAvatars show different responses to radiation combined with metformin

**Figure 3:**
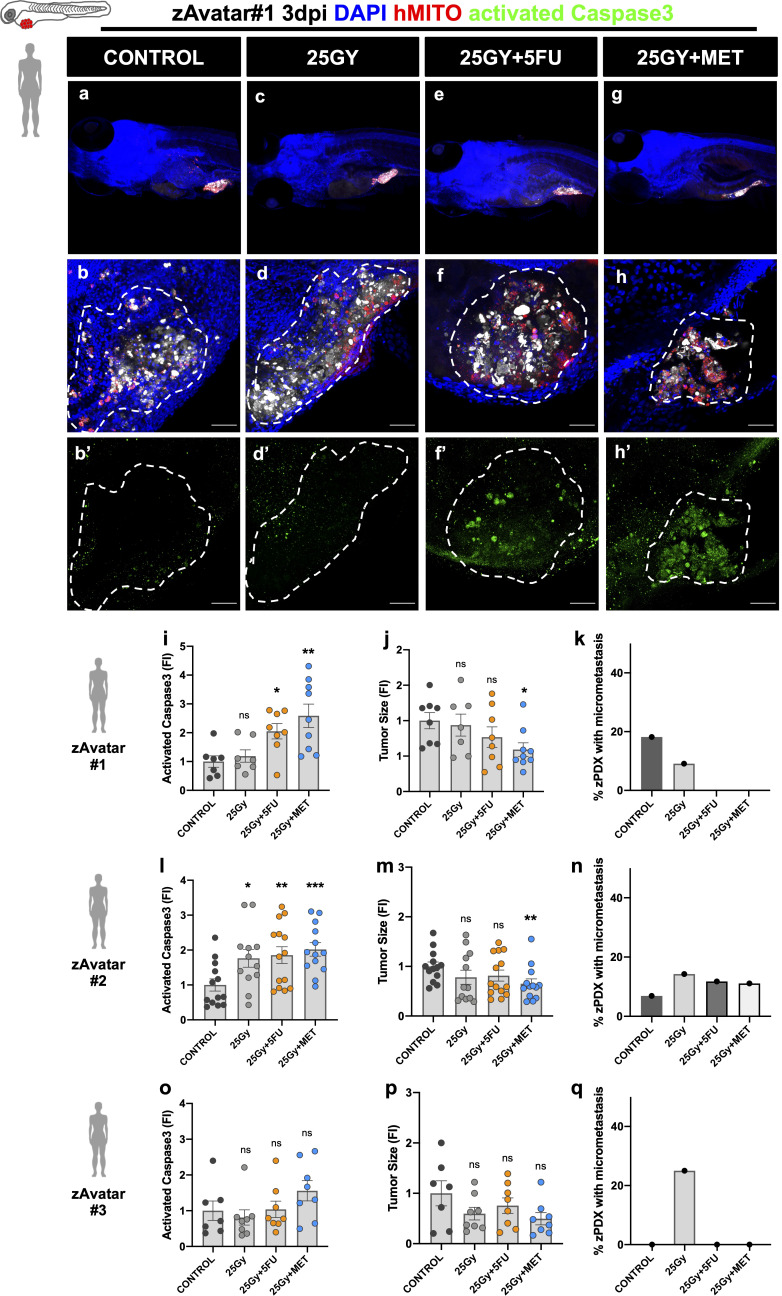
zAvatars were generated from rectum cancer surgical resected samples. One day after injection zAvatars were submitted to 25Gy radiation dose **(C, D, D’)**, 25Gy+5FU **(E, F, F’)** or 25Gy+MET **(G, H, H’)** regimens, and compared with control **(A, B, B’)** At 3dpi, apoptosis **(I, L, O)**, tumor size **(J, M, P)** and metastatic potential **(K, N, Q)** were analyzed. Tumor cells are labeled in white (cell tracker Deep Red), activated Caspase3 in green **(B’–H’)**, DAPI in blue and human-mitochondria marker in red. Images correspond to zAvatar#1, and quantifications are shown for zAvatar#1, #2 and #3. Data is expressed as mean ± SEM and each dot represents a xenograft. Dashed white line is delimitating the tumor of each xenograft. Scale bars represent 50µm. Statistical results: (ns) > 0.05, *P≦0.05, **P≦0.01, ***P≦0.001. Results were compared with control with exception of those that have a bar to indicate the compared groups.

**Figure 4:**
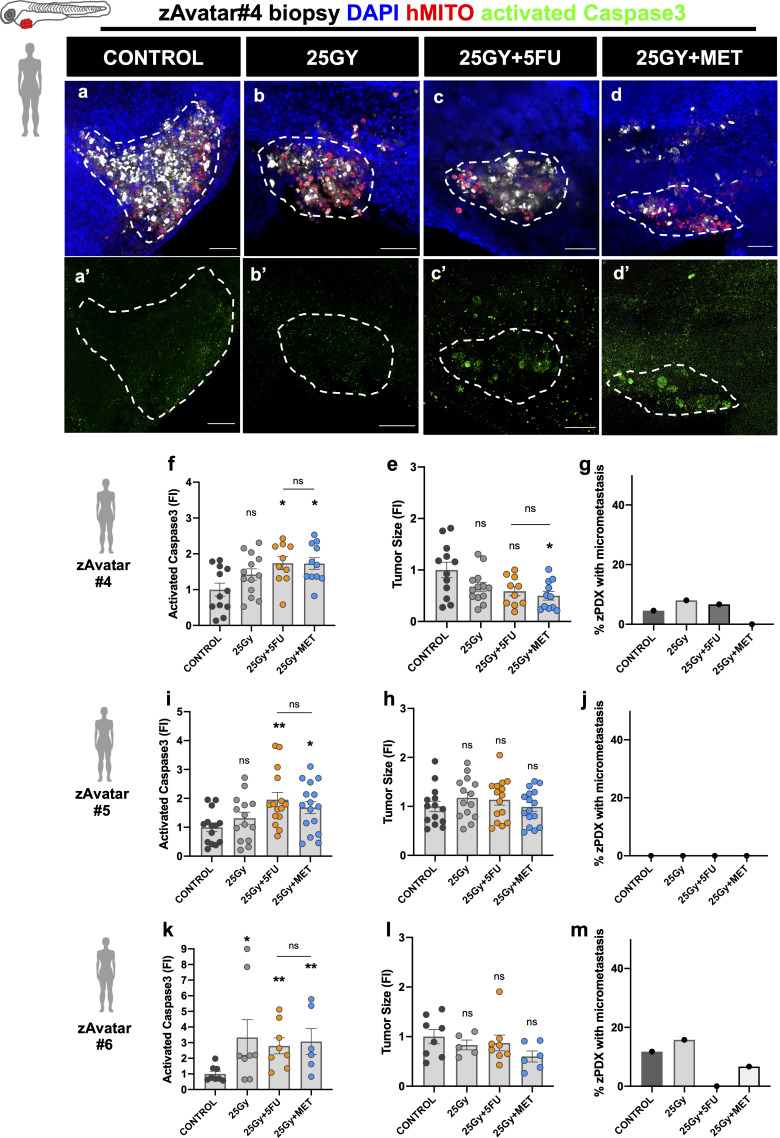
zAvatars were generated from naive rectal cancer biopsies. One day after injection zAvatars were submitted to 25Gy radiation dose **(B, B’)**, 25Gy+5FU **(C, C’)** or 25Gy+MET **(D, D’)** regimens, and compared with control **(A, A’)** At 3dpi, apoptosis **(F, I, K)**, tumor size **(E, H, L)** and metastatic potential **(G, J, M)** were analyzed. Tumor cells are labeled in white (Deep Red), activated Caspase3 in green **(A’–D’)**, DAPI in blue and human-mitochondria marker in red. Images correspond to zAvatar#4, and quantifications are shown for zAvatar#4, #5 and #6. Data is expressed as mean ± SEM and each dot represents a xenograft. Dashed white line is delimitating the tumor of each xenograft. Scale bars represent 50µm. Statistical results: (ns) > 0.05, *P≦0.05, **P≦0.01. Results were compared with control with exception of those that have a bar to indicate the compared groups.

Next, we used surgical resected rectal cancer samples (**Figure 3**) and rectum diagnostic biopsies obtained through endoscopy before neoadjuvant treatment (**Figure 4** and **Supplementary Figures S5**, **6**). Both were used without *in vitro* expansion to generate zebrafish patient derived xenografts (zPDX or zAvatars) (**Table 1**). We first used surgical resected rectal cancer samples to optimize and test the feasibility of our protocol, and only after, we proceeded to the biopsy samples, which due to the small amount of tissue becomes a more challenging technique. Since we do not amplify the tumor samples to reduce caveats of *in vitro* selection and time, which is crucial for decision making, the restrict amount of patient tissue is the major limitation of our assay. This is why it was not possible to test 5FU and MET alone. Nevertheless, it is in this setting – diagnostic biopsies – that the neoadjuvant options of treatment need to be tested prior to treatment for a future personalized management of the disease.

**Table 1:** Histopathological characterization of samples from rectal cancer patients included in the study.

Patient	Sample	Clinical Stage (before surgery)	Neoadjuvant treatment	Pathological Stage (after surgery)	P53	KRAS
**#1**	Surgical specimen	T3 N0 CRM- EMVI-	na	pT3N0	OE	wt
**#2**	Surgical specimen	T3 N0 CRM- EMVI-	na	pT3N0	OE	mut
**#3**	Surgical specimen	T4 N1 CRM+ EMVI+	scRT	ypT3 N1b	wt	wt
**#4**	Endoscopic biopsy	T3b N0 CRM- EMVI+	scRT	W&W	wt	mut
**#5**	Endoscopic biopsy	T2 N1 CRM- EMVI-	LC-CRT	W&W	wt	wt
**#6**	Endoscopic biopsy	T3b N2b CRM- EMVI-	na	pT3N0	OE	mut

scRT, short-course radiotherapy; LC-CRT, long course chemo-radiotherapy; W&W, watch & wait strategy; na, not-applied; mut, mutated; wt, wild-type; OE, overexpression (suggestive of a mutated protein); CRM, Circumferential Resection Margin; EMVI, Extramural Venous Invasion.

Patient samples ranged from T2 to T4 tumor clinical stages. Analysis of the KRAS and P53 revealed a diversity of combinations of KRAS and P53 status (mutated/wild-type) (see **Table 1** and **Supplementary Figure S7**). Samples were prepared for injection (see material and methods) and zAvatars were subjected to 4 different conditions at 1dpi, as previously described, and treated for 2 consecutive days. The percentages of implantation for all zAvatars are shown in **Supplementary Figure S3**.

**Figure 3** shows results obtained with surgical resected rectal cancer samples from 3 patients (#1, #2, and #3). Two of the surgical samples were naïve (#1, #2) but one had been previously treated with scRT before surgery (#3). Representative images are shown for zAvatar#1 (**Figures 3A–H**), including zoom out images (**Figures 3A–G**), and representative images of zAvatar #2, and #3 are shown in **Supplementary Figure S5**. **Figure 4** refers to zAvatars generated from rectal cancer biopsies obtained by endoscopy (#4, #5, and #6). All the endoscopic diagnostic biopsies were naïve. After clinical evaluation, two of the patients underwent radiotherapy treatments, scRT (#4) and LC-CRT (#5), whereas the third patient went directly to surgery (#6). Representative images are shown for zAvatar#4 (**Figures 4A–D**) and zAvatar#5 and zAvatar#6 are shown in **Supplementary Figure S6**. We analyzed induction of apoptosis and tumor shrinkage upon treatment, as well as the incidence of micrometastasis (percentage of zAvatars that presented micrometastasis in the tail, eye or gills; **Figure 3** and **Supplementary Figure S4**).

Overall, taking into account the induction of apoptosis (**Figures 3I, L, O**, **4F, I, K**), our results show that MET was able to sensitize tumor cells to RT to a similar extent as 5FU (25Gy+5FU and 25Gy+MET), with exception of one patient whose tumor cells appeared to be resistant (Patient#3). Of note, this patient presented a more advanced clinical stage and underwent radiotherapy before surgery, suggesting that we may have received radiation resistant clones, supporting our results that show resistance to radiotherapy (**Figure 3O**).

The impact of treatments on tumor size was not so evident (**Figures 3J, M, P**, **4E, H, L**), but this is not surprising given the short time assay and our previous work showing that induction of apoptosis is the readout that best correlates with patient’s clinical response (18, 19).

Regarding the impact on the metastatic potential, we could not find a trend, but in some patients 25Gy+MET was able to impair the metastatic spread of the tumor cells (zAvatar#1, #4 and #6). Also, zAvatars#1 exhibited ~20% of micrometastasis in control, which may constitute a “red alert” for the respective patient, requiring a more thorough follow up (**Figure 3Q**). Interestingly, the P53 status seems to correlate with a higher basal metastatic potential in control conditions (zAvatar#1, #2 and #6, without treatment).

Altogether, our results with zAvatars show that responses to treatment are heterogeneous and highly dependent on each patient’s tumor cells, not correlating with either the P53 or the KRAS status or their combination, in our small sample. Importantly, radiation combined with metformin was never inferior to the classical combination of radiation with 5FU, confirming the promising power of this less toxic approach for patients with rectal cancer.

## Discussion

Neoadjuvant chemoradiation is part of the standard of care for selected patients with rectal cancer, leading to significant tumor regression and sometimes achieving complete tumor responses. Increase of radiation therapy doses and/or the addition of more cycles of chemotherapy have been associated with higher tumor response rates. However, these strategies are associated with higher grades of toxicity (11, 12). Therefore, alternative regimens that improve tumor response rates without increasing treatment related morbidity are needed. In this context, the use of a well-tolerated radiosensitizing drug could be a perfect solution.

Metformin (MET) is a first line medication for the treatment of type II diabetes, presenting a well tolerable and a very low toxicity profile (27). Epidemiologic findings have demonstrated that metformin can reduce the risk of cancer in patients with type 2 diabetes (28–30). Moreover, a recent meta-analysis demonstrated the potential synergistic anti-tumor effects of metformin and radiotherapy on treatment of patients with cancer and type 2 diabetes (30). Specifically, in rectal cancer, a retrospective study showed an association of metformin use and improved tumor response to neoadjuvant chemoradiation (14). Furthermore, diabetic patients actively treated with metformin and affected with locally advanced rectal cancer (LARC) present higher rates of downstaging and pathological complete response (pCR) after CRT compared to those patients with LARC not taking metformin (31). In addition, it has been shown that metformin enhances tumor response of radiation therapy in several preclinical animal models using cell lines of lung, prostate and colon cancers (32–34). However, to our knowledge no studies have been performed with rectal patient samples.

Moreover, some other retrospective studies describe contradictory findings, where metformin were not protective or conferred any benefit in CRC (35–37). In most of these studies the dose and duration of metformin treatment were not known, probably contributing to the heterogeneity of the results (28, 30).

Therefore, pre-clinical studies using patient tumor samples and a randomized clinical trial where tumor stages, doses and treatment duration are controlled are essential to clarify the therapeutic effectiveness of metformin for the treatment of CRC (38).

In this work, we addressed the first issue – the use of patient tumor samples, without *in vitro* expansion. We tested whether we could detect the radiosensitizing effect of MET in the zebrafish xenograft model using patient samples xenografts (zPDX or zAvatars) and compare it with the standard 5FU. We first used two isogenic CRC cell lines (18, 24): HCT116 tumor cells harbor a KRASG13D mutation, rendering them highly proliferative and radiosensitive, and the isogenic KRASWT Hke3, that is less proliferative and is radioresistant (19). Our results show that MET has a similar radiosensitizer effect as 5FU in HCT116 xenografts. A recent publication showed similar effects in tumor growth impairment between RT/Metformin and RT/5FU, using a mouse model injected with SW480 CRC cell line (15). Interestingly, in Hke3 xenografts, MET can sensitize tumor cells to RT more efficiently than 5FU, constituting the only effective treatment for these radioresistant cells. Although the impact of KRAS mutations on radiosensitivity and MET is not clear, it has been shown that KRAS can influence and rewire the metabolic state of cancer cells and, therefore, could contribute to the different sensitivities to MET (39).

Next, we generated zAvatars from rectal tumor resected samples and diagnostic biopsies to test whether we could observe the radiosensitive effects of MET in patient samples. Our experiments with zAvatars demonstrate that response to the treatments tested are diverse and dependent of each patient’s tumor cells, but in general MET was able to sensitize tumor cells to RT to a similar extent as 5FU. Thus, the same radiosensitive effect could be achieved without chemotherapy-related toxicity. Nevertheless, there was one case where the tumor was resistant to all treatments, which corresponded to a surgical sample of a patient that was previously treated with scRT.

Patient samples used had different tumor stages (T2 to T4) and a variety of P53 and KRAS status. However, we could not detect any correlation between the P53 or the KRAS status or their combination with MET sensitivity, in our small sample. This suggests, like in most therapies, that there must be many more mutations and combinations (that we did not access) that contribute to the overall sensitivity. Genetic diversity influences multiple tumor phenotypes, like activation of signaling pathways, migration, metastization, senescence, metabolism, and ultimately impact on the sensitivity to treatment (16, 40). An extreme example shows that identical CRC cells, sharing the same genome, may have distinctive sensitivities to different therapies; indicating that besides the genetic heterogeneity, other factors like the environment/metabolism and epigenetics impact on therapy response (16, 41). This highlights the need for a functional test where tumor cells are directly challenged with therapies to access their sensitivity.

The exact mechanism of metformin anticancer action is not fully understood. There is substantial evidence indicating that metformin activates AMP-activated protein kinase (AMPK), inhibits mTOR-dependent translation initiation and affects cancer cell metabolism (42, 43). Also, when combined with radiation the mechanism of action has not been elucidated. Metformin cellular target is mitochondrial complex I, causing a severe depletion in ATP, increasing the production of reactive oxygen species and decreasing glutathione levels, exacerbating DNA damage (27, 30, 44). Other studies have suggested that MET can also reduce the expression levels of DNA repair enzymes and, consequently, impair DNA repair (45). In addition, MET is a potent modulator of glucose metabolism and potentially impacts on cancer progression (46). Although we did not address the mechanism of action of MET, the zebrafish xenograft model could be used in the future to test several hypotheses, for instance by adding high glucose concentrations (47) to the E3 medium or by treating xenografts with compound C, a AMPK inhibitor (48).

In conclusion, our work shows a powerful radiosensitive effect of MET *in vivo* using rectal cancer patient samples and, therefore, confirms that MET is a promising alternative to the standard 5FU protocol, which can be crucial for frail/elderly patients. Furthermore, zAvatars could be used to predict individual response prior to neoadjuvant therapy to tailor treatment in a more personalized manner, avoiding unnecessary toxicities.

## Data availability statement

The raw data supporting the conclusions of this article will be made available by the authors, without undue reservation.

## Ethics statement

The studies involving human participants were reviewed and approved by Ethics Committee of the Champalimaud Foundation. Rectal cancer patient samples were provided by Champalimaud Clinical Center’s (CCC) Digestive Unit. The patients/participants provided their written informed consent to participate in this study. The animal study was reviewed and approved by Ethical Committee of Champalimaud Foundation and Portuguese institutional organizations: ORBEA (Animal Welfare and Ethics Body) and DGAV (Directorate General for Food and Veterinary).

## Author contributions

RF, BC and LF conceptualized the research. RF supervised the research. BC performed research. LF, OP and IS participated in selecting and analyzing patient data. RR-T, MC-M and AP provided patient tumor samples. BC, LF and RF wrote the paper. RF provided funding. All authors contributed with the critical reading of the manuscript. All authors have read and agreed to the published version of the manuscript.

## Funding

We are grateful to Champalimaud Foundation, Congento (LISBOA-01-0145-FEDER-022170, co-financed by FCT/Lisboa2020) and FCT-PTDC/MEC-ONC/31627/2017 for funding.

## Acknowledgments

We would like to thank the patients who participated in the study, the Champalimaud Digestive Unit (Ignacio Herrando, Pedro Vieira, José Azevedo, Hugo Domingos), the Champalimaud Radiation Unit (Carlo Greco, Sandra Vieira, Joep Stroom, Maria João Cardoso, Ruben Coutinho), the Champalimaud Foundation Biobank (CFB) and the Champalimaud Fish Platform.

## Conflict of interest

The authors declare that the research was conducted in the absence of any commercial or financial relationships that could be construed as a potential conflict of interest.

## Publisher’s note

All claims expressed in this article are solely those of the authors and do not necessarily represent those of their affiliated organizations, or those of the publisher, the editors and the reviewers. Any product that may be evaluated in this article, or claim that may be made by its manufacturer, is not guaranteed or endorsed by the publisher.

## Abbreviations

5FU, 5-Fluouracil; AMPK, AMP-activated protein kinase; CRC, Colorectal cancer; dpf, days post fertilization; LARC, Locally advanced rectal cancer; MET, Metformin; mTOR, Mammalian target of rapamycin; nCRT, Neoadjuvant chemoradiation; mut, mutated; pCR, Pathological complete response; PVS, Periviteline space; RT, Radiotherapy; TME, Total mesorectal excision; wt, wild-type; zAvatar, Zebrafish xenograft model.
