# Supplementary material for: Zebrafish Avatars of rectal cancer patients validate the radiosensitive effect of metformin
Source: Front Oncol. 2022 Sep 28;12:862889. doi: 10.3389/fonc.2022.862889 (PMC9554544; doi:10.3389/fonc.2022.862889)
Supplement: Supplementary file 1 [file DataSheet_1.zip › Table S1.DOCX]

**Table S1:** Composition of media used for the generation of zAvatars.

|  | Reagent | Supplier | Final concentration |
| --- | --- | --- | --- |
| Collection medium | Advanced DMEM/F-12 | Gibco | - |
|  | Penicillin-Streptomycin | Sigma-Aldrich | 100U/mL |
|  | Amphotericin B solution | Sigma-Aldrich | 100μg/mL |
|  | Kanamycin solution | Sigma-Aldrich | 100μg/mL |
|  | Gentamicin solution | Sigma-Aldrich | 500μg/mL |
|  | Primocin | Invivogen | 100µg/mL |
| Mix 1 | DMEM F12 | Gibco | - |
|  | HEPES | ThermoFisher | 1% |
|  | Glutagro supplement | Corning | 1% |
|  | B27 | ThermoFisher | 1x |
|  | FBS (fetal bovine serum) | Sigma-Aldrich | 10% |
|  | Primocin | Invivogen | 100µg/mL |
|  | Anoikis inhibitor | Sigma-Aldrich | 10µM |
|  | Putrescine | Sigma-Aldrich | 10µg/mL |
|  | Epidermal Growth Factor (EGF) | PeproTech | 50ng/mL |
|  | N-acetylcysteine | Sigma-Aldrich | 1mM |
|  | Insulin-transferrin-selenium | Corning | 1% |
|  | Nicotinamide | Sigma-Aldrich | 10mM |
| Mix2 | Liberase TM | Roche | 50ug/mL |
|  | DNase | ThermoFisher | 5U/mL |
|  | Cell tracker Deep Red | Life Technologies | 1µL/mL |
