# Supplementary figures and images for: Zebrafish Avatars of rectal cancer patients validate the radiosensitive effect of metformin
Source: Front Oncol. 2022 Sep 28;12:862889. doi: 10.3389/fonc.2022.862889 (PMC9554544; doi:10.3389/fonc.2022.862889)

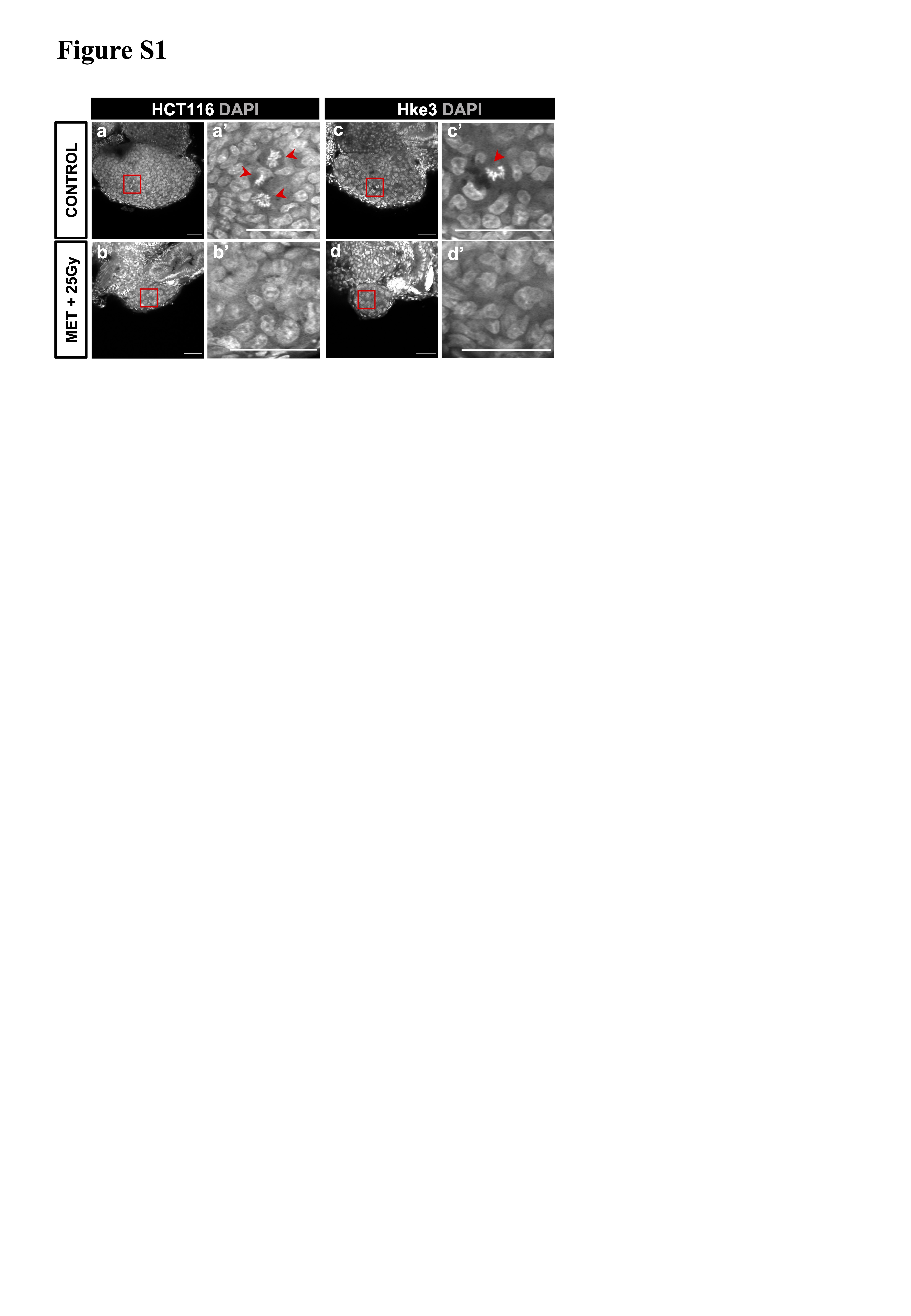

Supplement: Supplementary file 1 [file DataSheet_1.zip › Figure S1.JPEG]

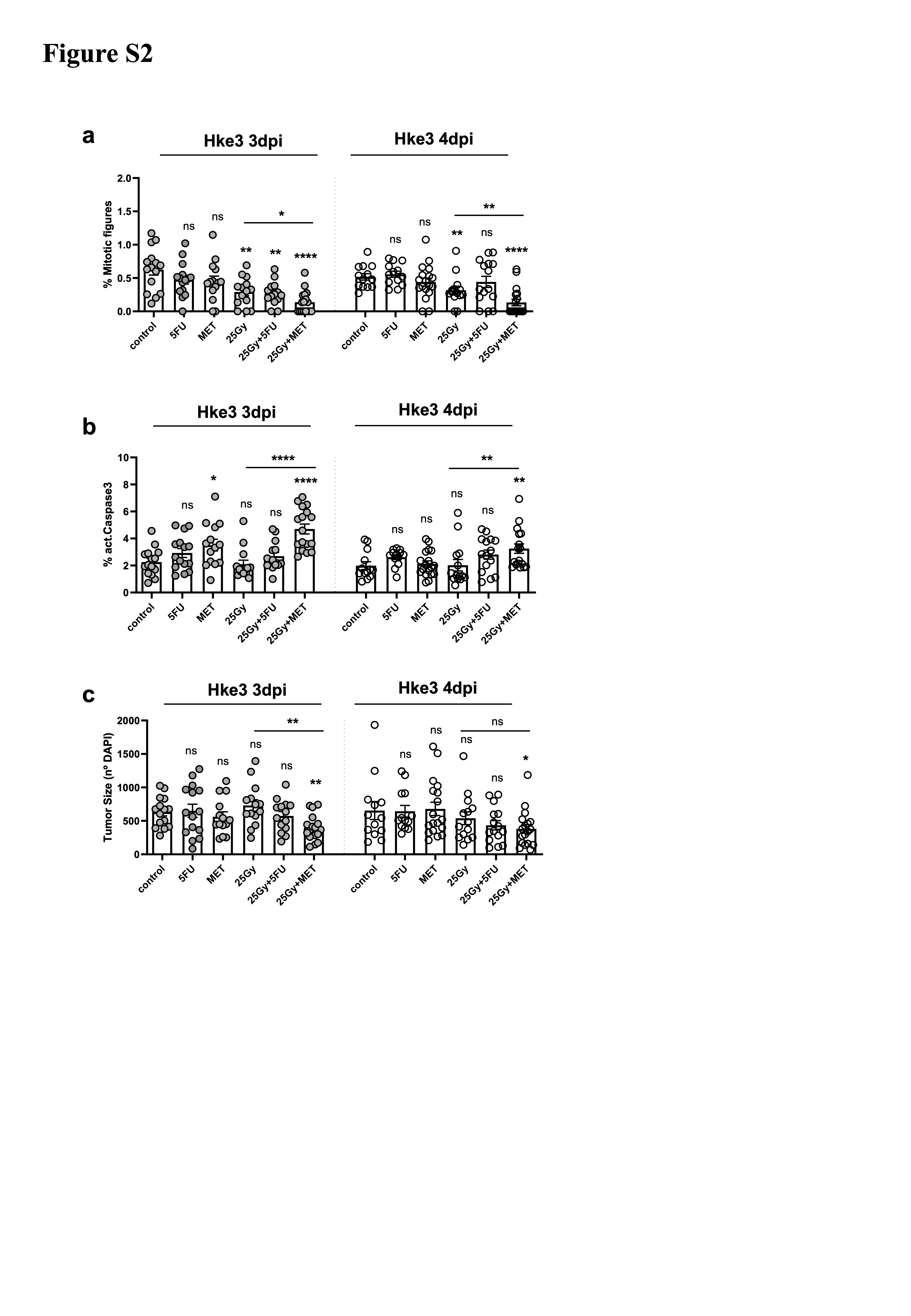

Supplement: Supplementary file 1 [file DataSheet_1.zip › Figure S2.JPEG]

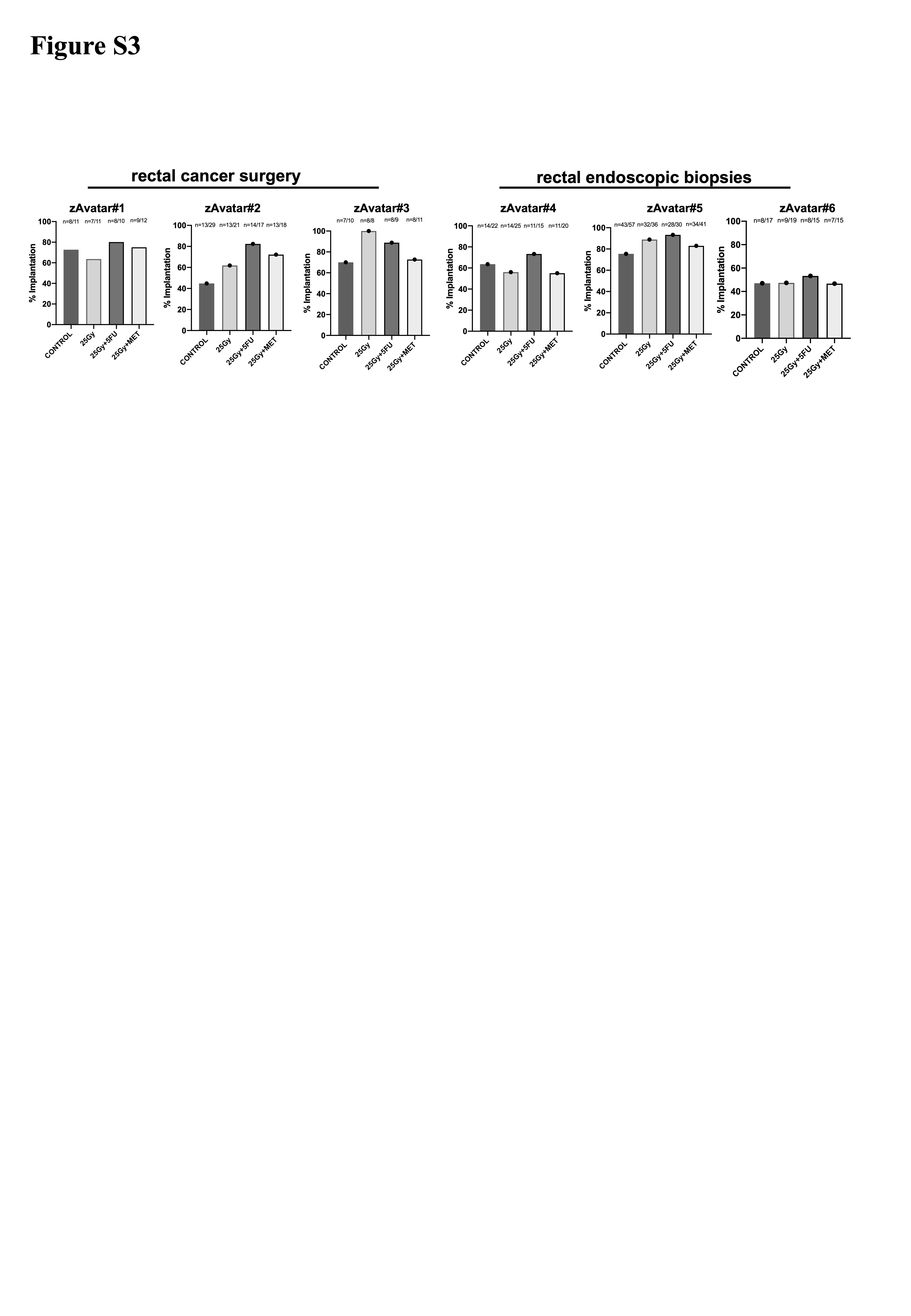

Supplement: Supplementary file 1 [file DataSheet_1.zip › Figure S3.JPEG]

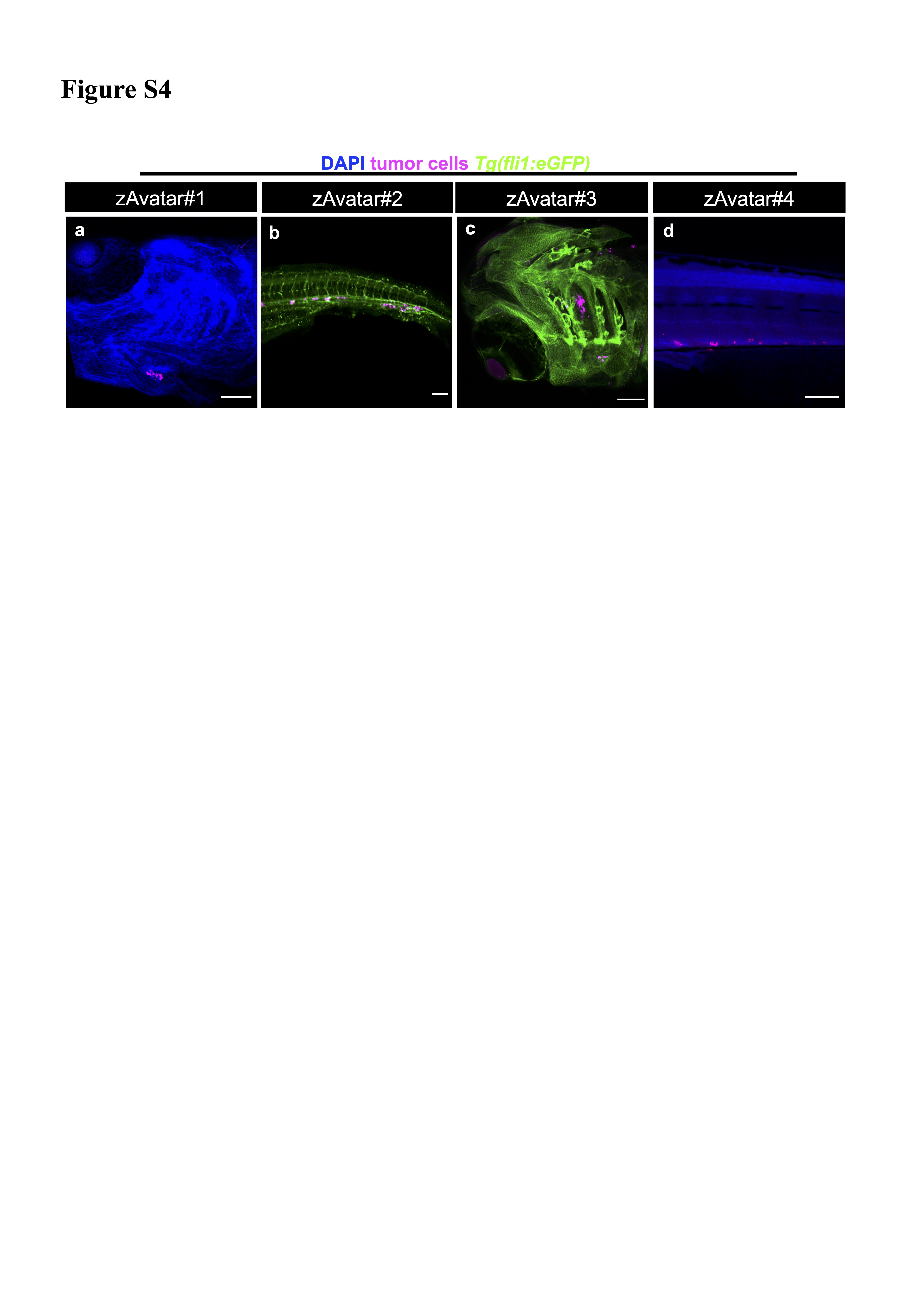

Supplement: Supplementary file 1 [file DataSheet_1.zip › Figure S4.JPEG]

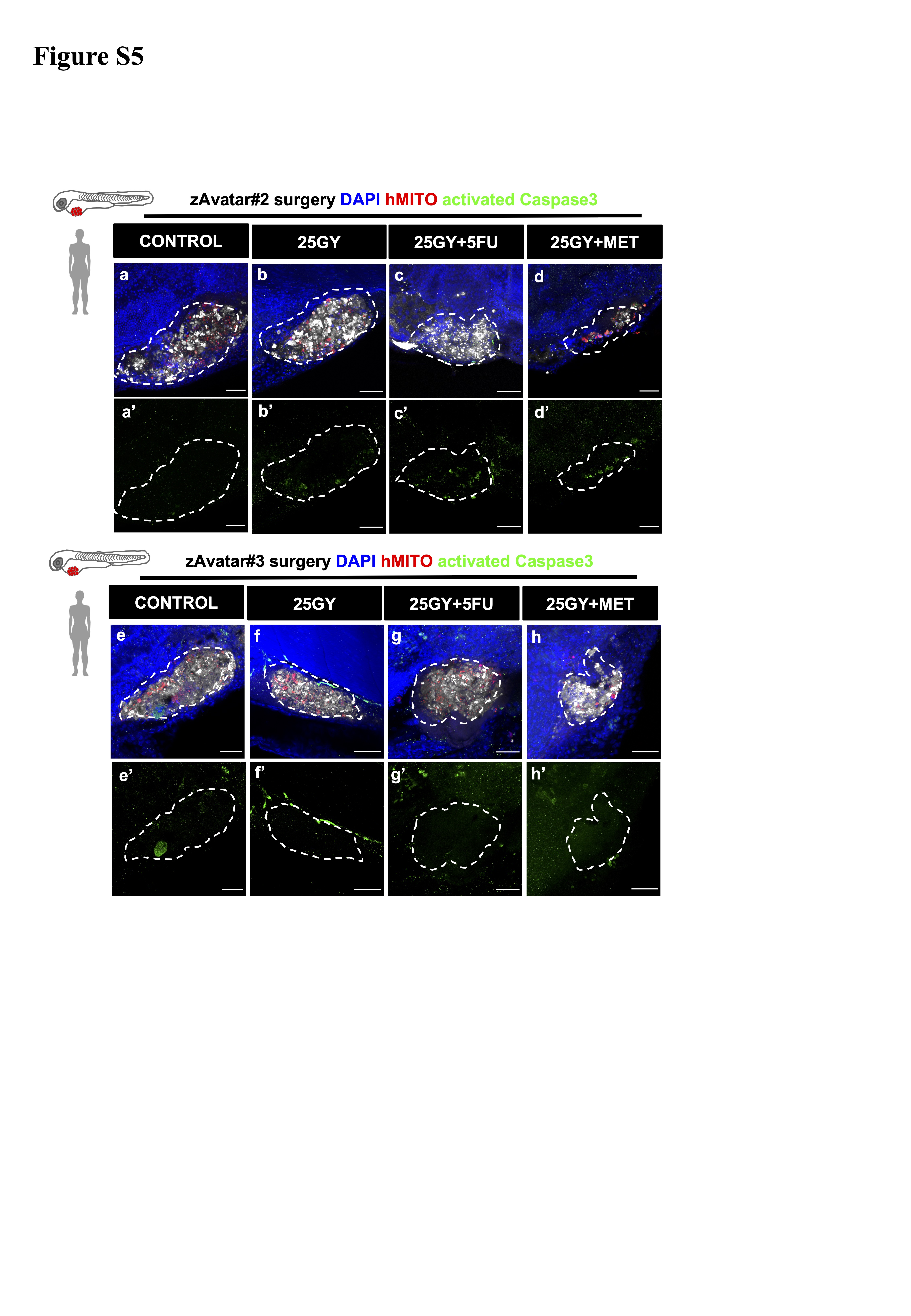

Supplement: Supplementary file 1 [file DataSheet_1.zip › Figure S5.JPEG]

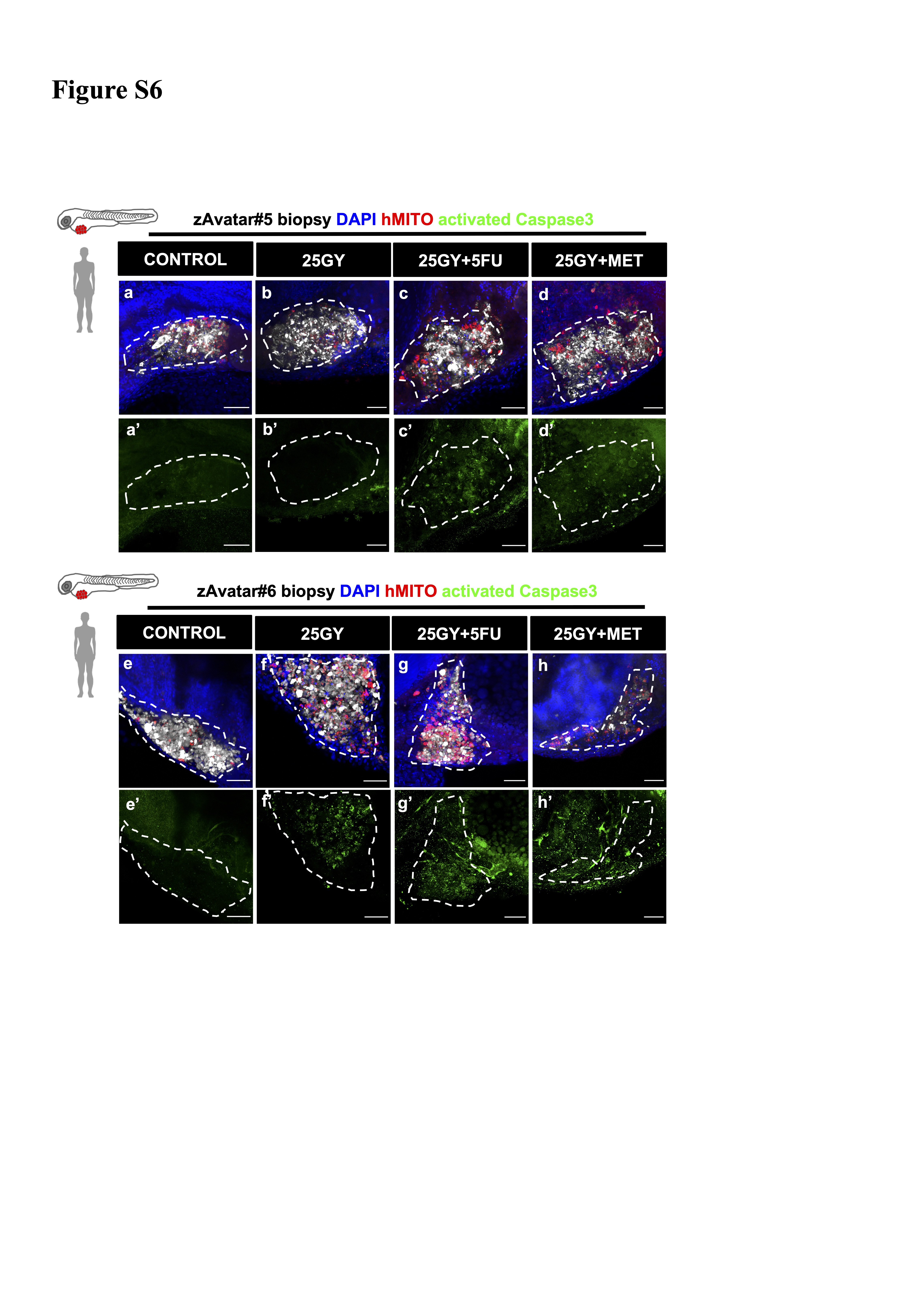

Supplement: Supplementary file 1 [file DataSheet_1.zip › Figure S6.JPEG]

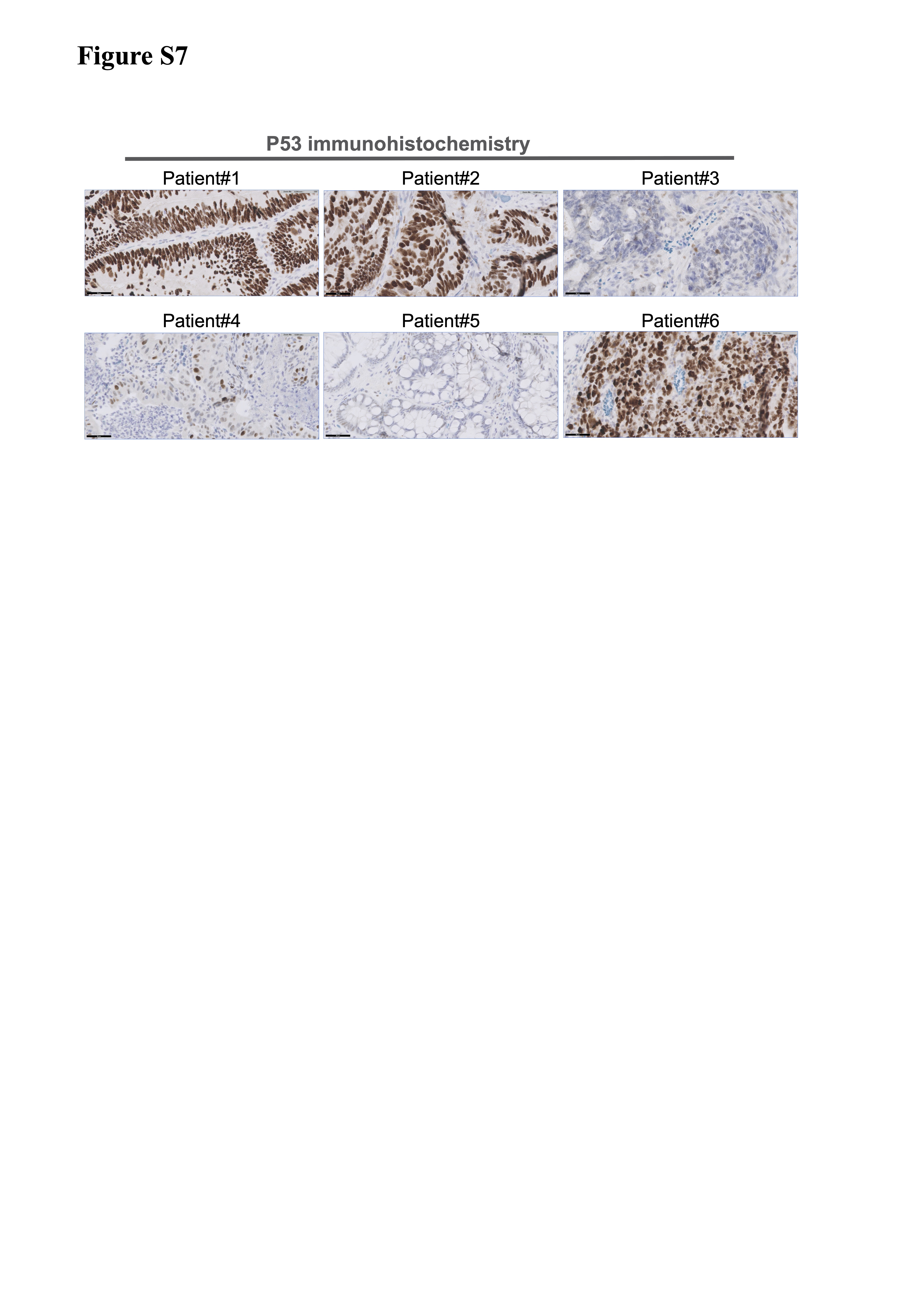

Supplement: Supplementary file 1 [file DataSheet_1.zip › Figure S7.JPEG]
